# The role of CT-scan in the diagnosis and prognosis of COVID-19 in a sample of Iraqi patients referred to Al-Diwaniyah Teaching Hospital, Iraq

**DOI:** 10.25122/jml-2023-0065

**Published:** 2023-07

**Authors:** Najat Adel Hashim, Khalil Kareem Al-Umeri, Amjaad Majeed Hameed

**Affiliations:** 1 Department of Surgery, College of Medicine, University of Al-Qadisiyah, Al Diwaniyah, Iraq

**Keywords:** COVID-19, CT-scan, CPAP, SARS-CoV-2

## Abstract

The respiratory system is the primary target of the SARS-CoV-2 virus, leading to clinical manifestations such as dry cough, fever, and shortness of breath. Other reported manifestations include generalized weakness, dizziness, headache, vomiting, and diarrhea. A chest CT scan is one of the best imaging tools to screen and diagnose COVID-19. This prospective observational study was conducted at Al-Diwaniyah Teaching Hospital in the mid-Euphrates region of Iraq to assess the prognostic role of chest CT examinations in COVID-19 patients between February 2020 and July the 15^th^, 2020. The study included 100 patients suspected of COVID-19 based on clinical features and microbiological investigations, comprising 72 males and 28 females aged between 30 and 55. All patients were SARS-CoV-2 PCR positive and had no history of chronic lung disease. We categorized patients into two groups based on changes in density and lesion area: patients with changes (n=55) and patients without changes (n=45). Furthermore, we divided all patients into three groups according to treatment requirements and symptom severity: group 1 included patients with severe symptoms who required CPAP and admission to the Respiratory Care Unit, group 2 consisted of patients with moderate symptoms who needed oxygen therapy and hospital admission, and group 3 comprised patients with mild symptoms who were treated as outpatients with medication. Upon correlating the change in density and lesion area with these groups, we observed that most patients with no changes were in group 1, while patients with changes were predominantly seen in group 2. Additionally, the ratio of lesion area in the mediastinal CT window to lung CT was identified as a potential prognostic factor for COVID-19 patients.

## INTRODUCTION

In December 2019, a series of acute atypical respiratory attacks occurred in Wuhan, a major industrial city in China. The disease rapidly spread to other areas, indicating a concerning outbreak [1]. Extensive medical investigations revealed that the attacks were caused by a novel coronavirus, named severe acute respiratory syndrome coronavirus 2 (SARS-CoV-2). This nomenclature was proposed because of the high similarity with SARS-CoV, the virus that caused acute respiratory distress syndrome (ARDS), and the high mortality rate during 2002 and 2003 [2]. Initially, the outbreak was thought to have a zoonotic mode of transmission via seafood, but subsequent evidence revealed that human-to-human transmission was primarily responsible for the rapid viral spread [3].

The constellation of signs and symptoms associated with SARS-CoV-2 was categorized as Coronavirus Disease 19 (COVID-19). Several weeks after the initial discovery, the World Health Organization (WHO) declared the outbreak a pandemic and almost all countries worldwide have reported cases [4, 5]. According to the Center for Systems Science and Engineering (CSSE) at Johns Hopkins University, as of April 7^th^, 2020, there have been approximately 1,400,000 reported cases globally [6].

Although several organ systems are also targeted by the SARS-CoV-2 virus, the virus principally attacks the respiratory system [1]. Clinical manifestations of lower respiratory tract involvement, such as dry cough, fever, and shortness of breath, have been reported in early cases discovered in China [7]. Other reported manifestations included generalized weakness, dizziness, headache, vomiting, and diarrhea [8]. The respiratory manifestations associated with SARS CoV-2 are highly variable, with mild and trivial features on one extreme and fatal ARDS on the other. The progression of disease in severe cases is exceptionally rapid, as ARDS has been shown to develop within 9 days of the start of symptoms [7]. The overall mortality rate of COVID-19 is approximately 1% in the general population and 13% in hospitalized patients [9]. It has been shown that the mortality rate is much lower in children but higher in the elderly population [10-12]. Supportive care is the main treatment strategy, as no specific antiviral agents have proven effective in clinical trials [10, 13, 14].

Chest computed tomography (CT) scan is considered one of the most valuable imaging tools for COVID-19 screening and diagnosis. In the early stages of the disease, chest imaging reveals interstitial changes and numerous plaque shadows, primarily located in the peripheral lung regions. Over time, these lesions merge, resulting in ground glass opacities and infiltrative shadows throughout both lungs. Lung consolidation happens in severe cases, appearing as “white lung,” rarely associated with mediastinal lymph node enlargement and pleural effusion [15].

This study was conducted in Al-Diwaniyah province, located in the mid-Euphrates region of Iraq, to evaluate the diagnostic and prognostic role of chest CT examinations in COVID-19 patients.

## MATERIAL AND METHODS

This observational prospective study was conducted at Al-Diwaniyah Teaching Hospital in Al-Diwaniyah Province, in the mid-Euphrates region of Iraq. The study started in February 2020 and concluded on July 15^th^, 2020. A total of 100 patients suspected of having COVID-19 based on clinical features and microbiological investigations were enrolled in the study. Among the participants, 72 were male and 28 were female. All participants had no previous history of chronic lung disease. SARS-CoV-2 polymerase chain reaction (PCR) testing confirmed the presence of the virus in all patients.

All patients underwent a comprehensive chest CT scan, which covered both lungs and the mediastinal region. The scan was performed from the lower neck to below the diaphragm with a single breath-hold technique. The images were acquired with a slice thickness of 1 mm. Radiological features of the disease, including lesion area and any changes in density or area between the lung window and mediastinum window, were evaluated. All data obtained were transferred to a spreadsheet in the Statistical Package for the Social Sciences (SPSS) software, version 23 (IBM, Chicago, USA). Quantitative variables were presented as mean, range, and standard deviation, while qualitative data were expressed as numbers and percentages. The association between qualitative variables was assessed using the chi-square test. A significance level of p≤0.05 was considered statistically significant.

## RESULTS

The study included 100 patients with signs and symptoms suggestive of COVID-19. The mean age of the patients was 42.53±7.82 years, ranging from 30 to 55 years. Among the participants, 72 (72.0%) were male, and 28 (28.0%) were female.

Table 1 displays the classification of patients based on the three main radiological CT-scan findings. Among the patients, 46 (46.0%) exhibited a diffused ground glass appearance, 32 (32.0%) showed peripheral consolidation, and 22 (33.0%) presented with a single nodule of consolidation with air.

**Table 1:** Classification of patients according to the three main radiological CT scan findings

CT scan findings	Number of cases (%)
Diffused ground glass appearance	46 (46.0 %)
Peripheral consolidation	32 (32.0 %)
Single nodule of consolidation with air	22 (33.0 %)

Table 2 compares the lesion's area in the lung and mediastinum windows, where significant variations were observed. The severity of the patient's symptoms was classified based on the type of treatment received, as shown in Table 3. Of the patients, 30 (30.0%) had mild disease (requiring medication), 44 (44.0%) had moderate disease (requiring medication with oxygen therapy), and 26 (9.1%) had severe disease (requiring medication and CPAP).

**Table 2:** Comparison of lesion area between lung window and mediastinum window

CT-scan findings	Change in density and area n=55	No change in density and area n = 45	p
Diffused ground glass appearance n=46	28 (50.9 %)	18 (40.0 %)	< 0.001 C HS
Multiple small patches of consolidation n=32	22 (40.0 %)	10 (22.2 %)	0.031 Y S
Single round pneumonia n=22	5 (9.1 %)	17 (37.8 %)	0.163 Y NS

n: number of cases; C: Chi-square test; Y: Yates correction; HS: highly significant at p≤0.01; S: significant at p≤0.05; NS: not significant at p>0.05

**Table 3:** Classification of symptoms severity based on the treatment received

CT-scan Findings	Treatment	Number of cases (%)
Total	Group 1 (medication)	30 (30.0 %)
	Group 2 (medication with oxygen therapy)	44 (44.0 %)
	Group 3 (medications and CPAP)	26 (9.1 %)

n: number of cases; CPAP: Continuous positive airway pressure

Treatment provided to the patients was primarily supportive, with some receiving no ventilator support and others receiving oxygen, continuous positive airway pressure (CPAP), or other modes of treatment. During the follow-up period, changes in both density and area were observed in some patients, while others showed no such changes. Consequently, the patients were categorized into two groups: those with changes (n=55) and those without changes (n=45). When considering all patients as a group, the need for oxygen, CPAP, and other treatments was significantly more frequent in patients without changes compared to those with changes (p<0.001). Similar observations were made when comparing patients with diffused ground glass appearance and multiple small patches of consolidation in terms of changes in radiologic area and density (p<0.05). However, no significant associations were found when considering patients with single-round pneumonia (p>0.05), as shown in Table 4.

**Table 4:** The association between changes in chest CT scan findings and treatment protocol used in patients with COVID-19 according to the severity of the clinical setting

CT-scan Findings	Treatment	Change in density and area n=55	No change in density and area n=45	p
Total	None	30 (54.5 %)	0 (0.0 %)	< 0.001 C HS
	Oxygen	20 (36.4 %)	24 (53.3 %)	
	CPAP and other	5 (9.1 %)	21 (46.7 %)	
Diffused ground glass appearance n=46	None	20 (71.4 %)	0 (0.0 %)	< 0.001 C HS
	Oxygen	7 (25.0 %)	10 (55.6 %)	
	CPAP and other	1 (3.6 %)	8 (44.4 %)	
Multiple small patches of consolidation n=32	None	10 (45.5 %)	0 (0.0 %)	0.031 Y S
	Oxygen	8 (36.4 %)	5 (50.0 %)	
	CPAP and other	4 (18.2 %)	5 (50.0 %)	
Single round pneumonia n=22	None	0 (0.0 %)	0 (0.0 %)	0.163 Y NS
	Oxygen	5 (100.0 %)	9 (52.9 %)	
	CPAP and other	0 (0.0 %)	8 (47.1 %)	

n: number of cases; CPAP: Continuous positive airway pressure; C: Chi-square test; Y: Yates correction; HS: highly significant at p≤0.01; S: significant at p≤0.05; NS: not significant at p>0.05

## DISCUSSION

Recent studies have shown common findings on lung CT scans obtained from patients with pneumonia attributed to COVID-19. Lung opacities, single or multiple, with ground glass appearance with or without consolidation, are often recognized [16]. These findings may be associated with thickening of blood vessels inside opacities. On the other hand, lesions are usually seen in multiple lobes and, in particular, the lower lobes. Another characteristic of these lesions is the peripheral location with or without a central location, considering that sole occurrence in a central location is rare [17]. Some other findings include interlobular septal thickening, a crazy-paving pattern, and air bronchograms. The observed changes in CT findings are variable and correlate with the stage of COVID-19 pneumonia [18]. Ground glass opacities are initially seen unilaterally or bilaterally associated with enlarged blood vessels. Focal consolidation may be associated with these opacities during the early stages. The lower lobe and sub-pleural areas (peripheral) are most often affected [19].

A proposed pathological mechanism for early peripheral involvement may be related to the behavior of the virus itself in that early involvement usually spreads from affected bronchioles to nearby parenchyma [20]. With further progress, there will be an appearance of new ground glass opacities, enlargement of previous ones, and involvement of more lung lobes. In addition, previous ground glass opacities may start to consolidate. This stage is critical and can be life-threatening, especially in patients with compromised immunity or inadequate treatment. CT scans may reveal diffuse opacity, often called “white lung manifestation” [21].

In patients with intact immune systems and optimal medical care, these CT findings resolve over time. The opacity gradually reduces and eventually disappears [22]. In this study, the main focus was to investigate the association between changes in lesion density and size and patient outcomes. Our findings indicate that the persistence of lesions is associated with a poor prognosis and a higher mortality rate compared to patients who show a continuous reduction in the size and density of lung lesions. Therefore, CT scans can be valuable for the initial diagnosis, monitoring the therapeutic response, and predicting the future outcomes of COVID-19 patients.

A meta-analysis [23] included 9907 confirmed COVID-19 cases and identified several common CT findings, including ground-glass opacities (77.18%), reticulations (46.24%), and air bronchograms (41.61%). Lesions were predominantly distributed bilaterally (75.72%) and peripherally (65.64%) [19]. Pre-existing lung diseases were present in a small percentage of cases (6.01%), while 8.20% (95%CI = 6.30–10.61) of patients had no abnormal findings [24].

According to reports, numerous bilateral ground-glass opacities (GGOs) with a predominately peripheral distribution are the typical chest CT finding of COVID-19 infection. Although these imaging features are thought to represent the majority of COVID-19 cases, different CT findings have been reported [25–27].

A recent meta-analysis of 4121 COVID-19 patients found comparatively similar results. The most frequent findings observed in their analysis were consolidation (32.0%), crazy-paving pattern (35.6%), air bronchogram (44.7%), and ground-glass opacities (68.1%). In comparison, lymphadenopathy and pleural effusion were identified in 5.4% and 5.3% of patients, respectively, while pleural thickening was present in 27.1% [28].

Generally speaking, the typical features in chest CT scans of patients with confirmed COVID-19 were comparable to those in patients with other viral cases of pneumonia, primarily severe acute respiratory syndrome and Middle East respiratory syndrome [29-32]. However, some investigations have also noted abnormal and unusual chest CT appearances. Because of this, medical professionals, especially radiologists, should be aware of the unusual results as well, especially in situations with pulmonary comorbidities such as lung cancer [33-35].

The most important limitation of the present study was that the research included only one center in Adiwaniyah province and that future multicenter studies are required to validate the results of the current study.

## CONCLUSION

The findings of this study suggest that the ratio of the area of the mediastinal CT window to the lung CT window can serve as a prognostic factor in patients with COVID-19. Regarding the size of the involved area, patients who presented with diffuse ground glass appearance lesions in the lung CT window and demonstrated a decrease in the area of the lesion in the mediastinum CT window had better prognoses compared to those with a fixed area of consolidation in both the lung and mediastinum CT windows. Regarding the density of the involved area, our study revealed that patients with faint density lesions in the lung CT window and decreased density within the mediastinum CT window had better prognoses than those with a fixed soft tissue density consolidation in the mediastinum CT window.
